# The SAH7 Homologue of the Allergen Ole e 1 Interacts with the Putative Stress Sensor SBP1 (Selenium-Binding Protein 1) in *Arabidopsis thaliana*

**DOI:** 10.3390/ijms24043580

**Published:** 2023-02-10

**Authors:** Irene Dervisi, Orfeas Petropoulos, Adamantia Agalou, Varvara Podia, Nikolaos Papandreou, Vassiliki A. Iconomidou, Kosmas Haralampidis, Andreas Roussis

**Affiliations:** 1 Department of Botany, Faculty of Biology, National and Kapodistrian University of Athens, 15784 Athens, Greece; 2 Laboratory of Toxicological Control of Pesticides, Scientific Directorate of Pesticides’ Control & Phytopharmacy, Benaki Phytopathological Institute (BPI), 8 Stefanou Delta Street, Kifissia, 14561 Athens, Greece; 3 Department of Cell Biology and Biophysics, Faculty of Biology, National and Kapodistrian University of Athens, 15784 Athens, Greece

**Keywords:** selenium binding protein 1, allergen SAH7, ABA, ROS, selenite, docking, Y2H, BiFC, protoplasts

## Abstract

In this study, we focused on a member of the Ole e 1 domain-containing family, *At*SAH7, in *Arabidopsis thaliana*. Our lab reports for the first time on this protein, *At*SAH7, that was found to interact with Selenium-binding protein 1 (*At*SBP1). We studied by GUS assisted promoter deletion analysis the expression pattern of *AtSAH7* and determined that the sequence 1420 bp upstream of the transcription start can act as a minimal promoter inducing expression in vasculature tissues. Moreover, mRNA levels of *AtSAH7* were acutely increased under selenite treatment in response to oxidative stress. We confirmed the aforementioned interaction in vivo, in silico and in planta. Following a bimolecular fluorescent complementation approach, we determined that the subcellular localization of the *At*SAH7 and the *AtSAH7*/*At*SBP1 interaction occur in the ER. Our results indicate the participation of *At*SAH7 in a biochemical network regulated by selenite, possibly associated with responses to ROS production.

## 1. Introduction

Allergens are proteins causing an IgE-mediated hypersensitivity response in humans. They are proteins with different molecular weights and chemical properties, with the Ole e 1 domain containing a protein family representing the major allergen group (Accession number: P19963) [1,2].

Ole e 1 protein was first characterized and purified from *Olea europaea* pollen [3] and named based on the International Union of Immunological Societies (IUIS) guidelines [4]. It is considered the main allergen causing a hypersensitivity reaction to olive pollen in more than 70% of patients [5]. Allergens comprising the Ole e 1 domain consisting of the conserved amino acid sequence E/Q/T-G-X-V-Y-C-D-T/N/P-C-R form the Ole e 1 protein family [2,6,7,8], divided in 109 sub-families based on amino sequence and structural similarities [2].

The Alché et al. study revealed the importance of Ole e I during pollen tube formation [9]; however, other studies in rice suggest that Ole e 1 domain-containing proteins may function in other tissues as well [7]. In more detail, the authors characterized allergens in rice using bioinformatic analysis, and then studied their expression by RT-PCR in different tissues, where some allergens were expressed apart from flowers in leaves and roots [7]. Interestingly, some allergens pertaining to expansins were root specific.

In *Arabidopsis thaliana* (L.) Heynh, there is an SBP (Selenium Binding Protein) gene family, comprised of *AtSBP1*, *AtSBP2* and *AtSBP3* [10,11]. *At*SBP1 was first described by Agalou and colleagues as a protein able to confer tolerance to selenium toxicity, as its overexpression showed enhanced tolerance to selenite, while plants with reduced expression levels were more sensitive to selenite [10]. Furthermore, *At*SBP1 interacts with other proteins related to vesicle trafficking, membrane synthesis and cellular redox control, including the allergen *At*SAH7 [12].

*At*SBP1′s possible function has been suggested to be part of a detoxification mechanism [11,13], as in mammals. There is evidence that this protein plays a role in cadmium (Cd) detoxification by direct binding to Cd^2+^ [14]. In addition, SBP1 overexpression enhanced tolerance against selenite [10] and cadmium [11], affecting the GSH levels [15].

Our group has demonstrated the in planta interactions of *At*SBP1 with the glutaredoxins *At*GRX14 and *At*GRX16 [16], phospholipase A1, *At*DALL3 [17], *At*GAPDH and *At*FBA [12]. More recently we have also demonstrated interactions of *At*SBP1 with the papain-like cysteine protease, *At*RD19c, that is probably involved in the activation of programmed cell death [18]. These results enhance our hypothesis that *At*SBP1 is a member of a protein network comprised of *At*SBP1, *At*GAPDH, *At*FBA, *At*GRX14, *At*GRX16, *At*DALL3 and *At*RD19c, as a detection and/or response system to stress. Finally, using the model system *Chlamydomonas reinhardtii*, we showed with combined global transcriptomic and metabolomic analysis that the *sbd1* (SBP) mutant exhibited a dramatic quenching of the molecular and biochemical responses upon H_2_O_2_-induced oxidative stress when compared to the wild-type. Our results indicated that *Cr*SBD1 represents a cell regulator, which is involved in the modulation of *Chlamydomonas reinhardtii*’s early responses to oxidative stress. We asserted that *Cr*SBD1 acts as a member of an extensive and conserved protein–protein interaction network in *C. reinhardtii*, including Fructose-bisphosphate aldolase 3, Cysteine endopeptidase 2, and Glutaredoxin 6 proteins, as indicated by yeast two-hybrid assays [19].

In this work, we focused on the *At*SAH7 (AT4G08685) allergen which is a member of the Ole e 1 allergen and extensin family. To our knowledge, *At*SAH7 has been characterized to date by our initial study that defined *At*SAH7 as an interacting candidate of *At*SBP1. The systematic and phylogenetic analysis of Jiménez-López for the Ole e I family classifies *At*SAH7 in the most extended family with 63 members, the Ole e I_48, which includes proteins analogous to C13 protein (corn allergen) [2]. Moreover, transcriptomic analysis showed that *At*SAH7 mRNA is transported to distant tissues [20].

In order to gain insight into *At*SAH7 function and contribute experimental data about this non-studied protein, we performed promoter and expression analysis during different developmental stages as well as under different chemical treatments. However, our main question was whether *At*SAH7 is capable of interacting with *At*SBP1, and if so where this interaction occurs.

## 2. Results and Discussion

### 2.1. Promoter Analysis of AtSAH7

**Figure 1:**
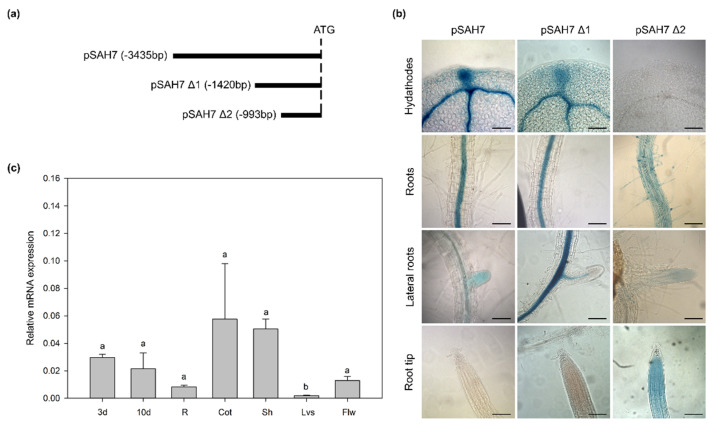
Promoter analysis and expression of *At*SAH7 in different developmental stages. (**a**) Schematic presentation of pSAH7 deletions. (**b**) Promoter analysis of *At*SAH7 in 6-day-old seedlings. pSAH7 fragments were transcriptionally fused to the GUS reporter gene and displayed different expression patterns. Bar 100 μm. (**c**) Relative expression analysis of *At*SAH7 in different developmental stages. Tissues examined: 3d: 3-day-old seedlings; 10d: 10-day-old seedlings; R: root; Cot: cotyledons; Sh: Shoots; Lvs: rosette leaves and Flw: flowers. Values ± SD were normalized to the mean of UBQ10 and represent the mean of three biological samples analyzed in triplicate. Significant differences at *p* ≤ 0.05 are indicated by letters a, b.

Aiming to investigate the transcriptional activity of the *AtSAH7* promoter, we generated stably transformed plants with promoter deletions. In more detail, a 3435 bp sequence upstream of the open reading frame of *AtSAH7* (pSAH7) was used, and then two successive 5′-end deletions were generated (pSAH7 Δ1: −1420 bp, pSAH7 Δ2: −993 bp) (Figure 1b). All fragments were fused to the *GUS* reporter gene. The expression patterns of the pSAH7 and pSAH7 Δ1 were quite similar, depicting staining in the hydathodes and vasculature of the cotyledons as well as in the vascular cylinder of the main root and in lateral roots (Figure 1a). In contrast to this expression pattern, the pSAH7 Δ2 fragment was lacking expression in the vascular tissues and GUS expression was observed only in root hairs and in root tips.

To investigate the differences between the observed patterns, we searched for promoter regulatory elements focusing on the sequence of pSAH7 Δ2 (−1420 bp to −993 bp). The possible promoter elements were estimated by the PlantCARE database [21] and are presented in Table S1. The predicted elements are related to light responses (GT1-motif, TCT-motif), drought, abscisic acid responses (Myc) and differentiation of the palisade mesophyll cells (HD-Zip 1). Amongst them, a possible element whose absence might affect GUS expression is the HD-Zip 1. The HD-ZIP transcriptional factors promote axial cell elongation and xylem differentiation [22,23,24]. Abolishment of the HD-Zip 1 promoter element in pSAH7 Δ2 could lead to the functional incapacity of this transcriptional factor, which determined the absence of GUS staining from vascular tissues (Figure 1a).

We can assume that the 1420 bp upstream region of *At*SAH7 ORF is sufficient to act as the minimal endogenous promoter of this gene. The expression pattern of *At*SBP1 was described before by Valassakis et al. [13], and some of the reported patterns overlap with these of *At*SAH7, indicating that the transcripts of *At*SAH7 and *At*SBP1 co-localize. In more detail, *At*SBP1 is induced in the vasculature tissue of roots, cotyledons and leaves as well as in hydathodes. Hydathodes are specialized organs that prevent the harmful effect of excess water and xylem sap [25,26,27]. Moreover, hydathodes are the auxin biosynthesis gene reservoir [28,29,30,31,32,33,34], while auxin has been observed in hydathodes via immunolocalization [29].

To further investigate the expression of *At*SAH7, its relative expression was measured in different developmental stages. The tissues examined were four day (4d) and ten day (10d) seedlings, roots (R), cotyledons (Cot), shoots (Sh) from ten day seedlings, rosette leaves (Lvs) and flowers (Flw) from four week old plants. Our analysis showed the constitutive expression of *AtSAH7*, with minima in leaves and statistical significance and a maximum in cotyledons, however, without statistical significance (Figure 1c).

The *AtSAH7* expression in vegetative cells is in line with previous studies on Ole e 1 domain-containing proteins (*AtPOE1*), showing root-specific patterns indicating additional developmental and physiological roles except in pollen tube formation [8]. Moreover, in spite of being allergens they play crucial role in root regeneration and seed and pollen germination [9,35]. Recent studies of a *Gerbera hybrid* Ole e 1 orthologue (*Gh*POE1) revealed its participation in leaf senescence [36].

### 2.2. Differential Expression of AtSAH7 under Se and Cd Exposure

**Figure 2:**
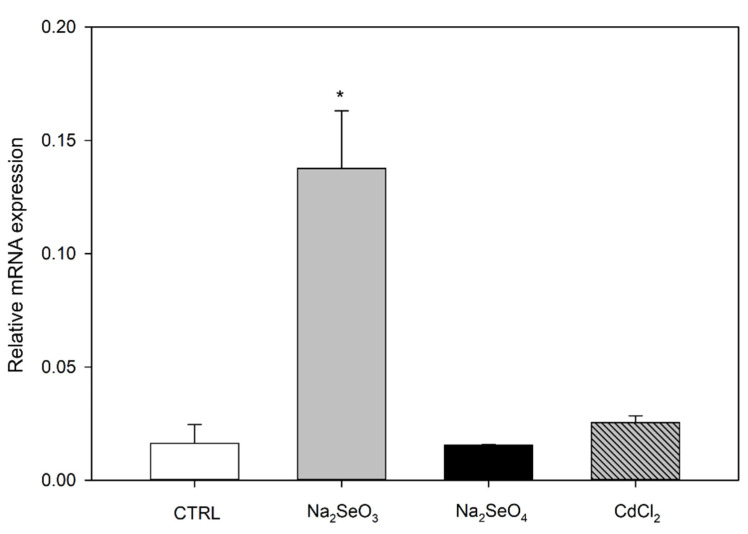
Relative expression levels of *At*SAH7 in Arabidopsis thaliana roots treated with selenium compounds (selenite, Na_2_SeO_3_; selenate, Na_2_SeO_4_) and cadmium (CdCl_2_). Significant upregulation of *At*SAH7 was observed after selenite (Na_2_SeO_3_) treatment. Values ± SD were normalized to the mean of UBQ10 and represent the mean of three biological samples analyzed in triplicate. Significant differences at *p* ≤ 0.05 are indicated by asterisks.

The relative expression of *AtSAH7* mRNA was measured in seedling roots after chemical treatment with different Se and Cd compounds. Our analysis demonstrated significant upregulation in the presence of Na_2_SeO_3_, while no notable change was observed in CdCl_2_ and Na_2_SeO_4_ (Figure 2). This is in contrast with *AtSBP1* expression under the same treatments that exhibited a reverse response in selenite and a remarkable upregulation in selenate and cadmium [17]. Our results indicate the participation of *At*SAH7 in a biochemical network regulated by selenite. Selenite, as well as selenate, leads to ROS accumulation in roots, as demonstrated by Valassakis et al. [13]. Thus, it is plausible to speculate that *At*SAH7 could be associated with responses to ROS production.

Additionally, in our promoter analysis, we found a Myc promoter element, which is described as an element responsible for abscisic acid responses. Cellular ROS levels are enhanced by abscisic acid (ABA) in *Arabidopsis* guard cells in order to promote stomatal closure [37,38]. Furthermore, ABA increases H_2_O_2_ levels in maize embryos and seedlings and in *Vicia faba* guard cells, a process that precedes stomatal closure [39,40,41,42]. At this point it is worth mentioning that we have previously demonstrated that *AtSBP1* is highly expressed in the guard cells of the stomata [13]. Moreover, ABA and ROS cross-talking is also observed during biotic and abiotic stress, as well in plant development and growth [43]. Another study focused on rice revealed that ABA produced during heat stress prevents the reduction of pollen viability and spikelet fertility [44]. These observations further support the role of ABA in ROS signaling. As *At*SAH7 is a pollen associated protein, it is not surprising that it is activated under ROS induction conditions, an observation in line with our data.

### 2.3. Subcellular Localization of AtSAH7

The subcellular localization of *At*SAH7 was studied in intact seven day old seedlings harboring the *At*SAH7 allergen fused to the *EYFP* reporter gene under the control of the 35S promoter. Seedlings were observed after Hoechst and propidium iodine (PI) staining.

**Figure 3:**
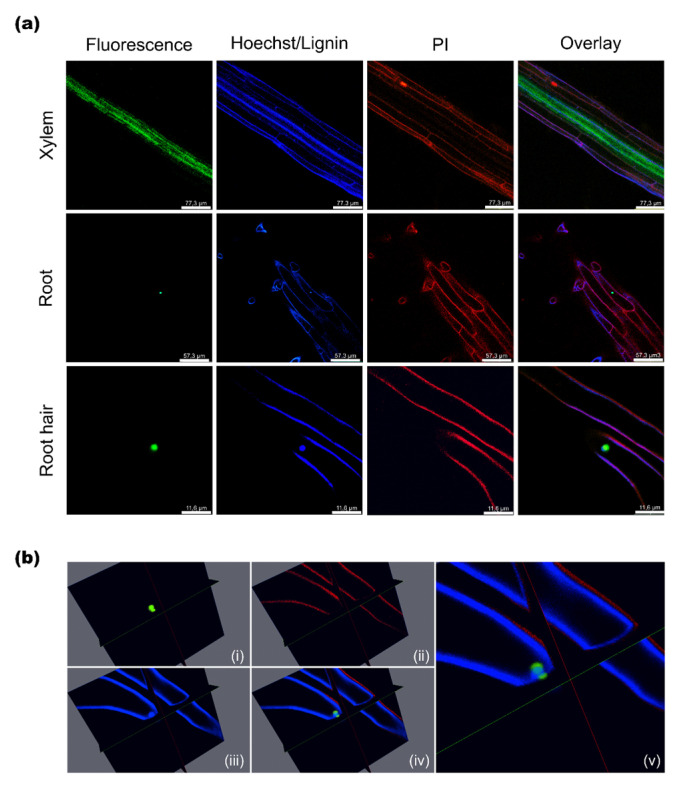
Subcellular localization of *At*SAH7 in 7-day-old roots in chimeric 35S:*At*SAH7-EYFP transgenic plants after PI and Hoechst 33258 staining. (**a**) Confocal imaging of xylem, root and root hair tissues. (**b**) 3D imaging of a root hair expressing *At*SAH7. (**i**) Fluorescence; (**ii**) PI; (**iii**) Hoechst 33258; (**iv**) Overlay; (**v**) Zoom of (**iv**) in the area of the signal.

In seven day seedlings, the fluorescent signal was observed only in root tissue and, specifically, in the xylem (Figure 3a), while in roots as well as in root hairs it was in spherical structures similar to the nucleus. Interestingly, at first look, *At*SAH7-EYFP seems to be co-localized with the Hoechst signal, which stains the nucleus; however, a closer and more detailed projection reveals that *At*SAH7 is not a nuclear protein. The *At*SAH7 signal, rather, surrounds the nucleus (Figure 3(bv)).

These findings are in line with our results from protoplast isolation and transfection. Protoplasts were isolated from stable mCherry-NLS Arabidopsis plants from mesophyll and roots. In both cases, protoplasts were transiently transfected with pSAT6/35S-*At*SAH7-EYFP. The signal observed in the fluorescent microscope was similar to a horseshoe that surrounds the nucleus (see Section 2.6).

In order to further investigate the subcellular localization potential of *AtSAH7*, we uploaded the protein sequence in the online prediction system MultiLoc2 (https://abi-services.cs.uni-tuebingen.de/multiloc2/webloc.cgi), accessed on 20 December 2022 [45]. When the predictor used for the analysis was MultiLoc2-LowRes, the results indicated that *At*SAH7 is a secretory pathway protein (secretory pathway: 0.97 cytoplasmic: 0.02 nuclear: 0.0 mitochondrial: 0.0 chloroplast: 0.0). Subsequently, we ran the analysis with the MultiLoc2-HighRes predictor and the results depicted ER: 0.44, extracellular: 0.17, plasma membrane: 0.17, Golgi apparatus: 0.1, vacuolar: 0.07, peroxisomal:, 0.01, mitochondrial: 0.01, cytoplasmic: 0.01, nuclear: 0.01, and chloroplast: 0.01. These analyses support that *At*SAH7 is targeted in the ER.

Subcellular localization in the ER by immunolocalization analyses for other proteins that are members of the Ole e 1 family, Pla1 and Ole e 1, has already been reported. More specifically, synthesis and storage of Ole e 1 occurs in the ER of vegetative cells in *Olea europaea* L. [46,47]. From the above, it is plausible to speculate that the observed structure that *At*SAH7 localizes in is either ER or ER-derived.

### 2.4. AtSAH7 Interacts Only with AtSBP1 in a Yeast Two-Hybrid Assay

**Figure 4:**
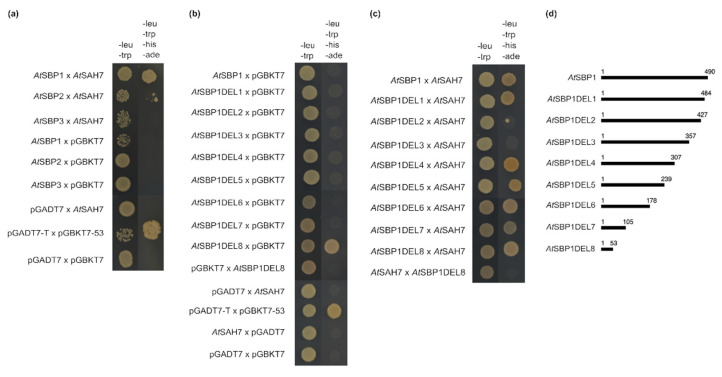
Yeast two-hybrid assay. (**a**) *At*SAH7 in vivo interactions with *At*SBPs. *At*SAH7 interacts only with *At*SBP1. (**b**–**d**) *At*SBP1 deletions examined for physical interaction with *At*SAH7. (**b**) Controls used for the deletion analysis. (**c**) Interaction analysis of *At*SBP1DELs with *At*SAH7. (**d**) Schematic presentation of *At*SBP1 truncations used. The first 53aa are important for the aforementioned interaction. To exclude false positives proper controls were used, including pGADT7 × pGBKT7 as a negative control and pGADT7-T × pGBKT7-53 as a positive control.

In order to verify the initial positive interaction of *At*SAH7 and *At*SBP1 that was previously reported by Agalou et al. [12], individual protein–protein interaction experiments were performed. Furthermore, the other two selenium-binding proteins (*At*SBP2 and *At*SBP3) were tested. The yeast two-hybrid assay confirmed a strong positive interaction only with *At*SBP1 (Figure 4a).

To determine the domains of *At*SBP1 that are responsible for binding *At*SAH7, we generated eight successive C-terminal deletions of *At*SBP1 and each of them was verified for its ability to interact with the allergen. Our analysis showed that the first 105aa harbor the full binding capacity to interact with *At*SAH7, whereas the region between 307aa and 484aa, when deleted, prevents the proper interaction (Figure 4b–d). This effect could be caused due to the misfolding of the *At*SBP1, thus concealing the responsible interaction sites. Moreover, *At*SBP1DEL8 proved in a previous study to be a self-activating construct [16] when the particular truncated version of *At*SBP1 is cloned in frame with the AD domain of the pGADT7. Therefore, we followed the strategy of domain swapping of the AD and BD domains in relation to *At*SBP1DEL8 and *At*SAH7, thus demonstrating that in this arrangement *At*SBP1DEL8 is no more self-activating, as shown in Figure 4b,c. It is noteworthy that the first 178aa are important for the interaction of *At*SBP1 with *At*GRXS14 [16], as well as with *At*DALL3 [17] and *At*RD19c [18].

### 2.5. Protein Molecular Modeling and Structural Prediction of Protein–Protein Interactions

The results of the yeast two-hybrid (Y2H) assays for the complex of *At*SBP1 and *At*SAH7 indicate that certain deletions of *At*SBP1 (specifically deletions 1, 4, 5, 6 and 7) interact with *At*SAH7. These observations drove us to conduct molecular docking experiments and molecular dynamics simulations in order to study the mode of interaction between *At*SBP1 and *At*SAH7.

**Figure 5:**
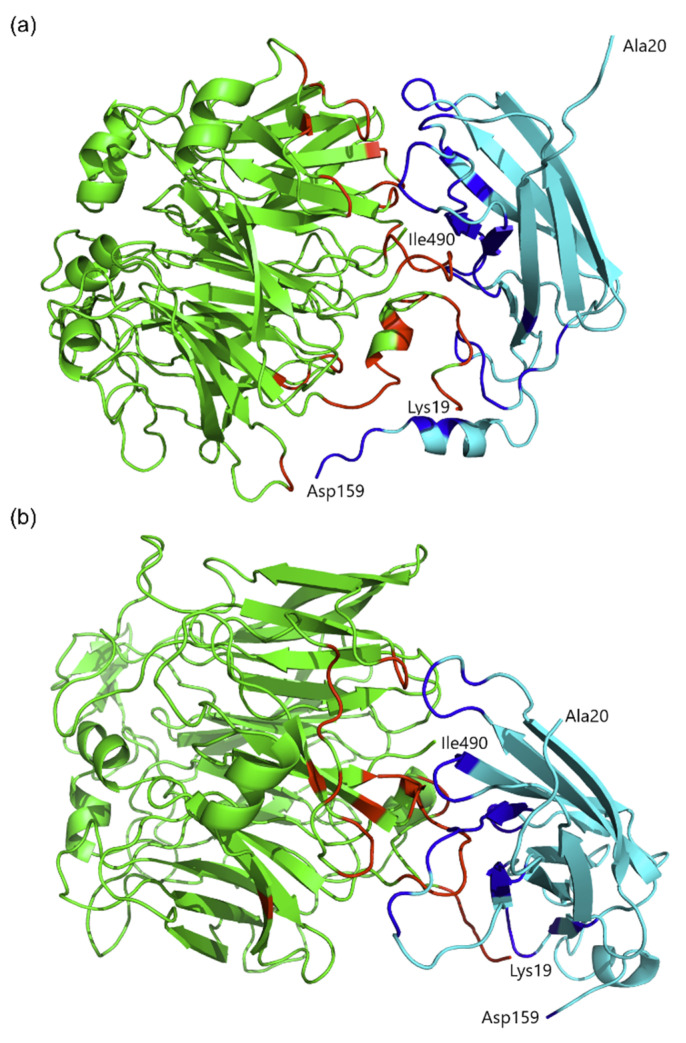
A cartoon representation of the predicted *At*SBP1–*At*SAH7 complex (colored in green and cyan, respectively) after the docking experiment (**a**) and the molecular dynamics simulation (**b**). The interfacing residues, calculated by PISA, are colored red (*At*SBP1).

Driven docking experiments were carried out utilizing the HADDOCK2.2 Web Server. The lack of experimentally determined structures for both proteins led us to retrieve their theoretical structural models from Alphafold DB, which contains models for the majority of the entries of Uniprot, predicted by the state-of-the-art software Alphafold. The predicted model of *At*SBP1 by Alphafold adopts the fold of a seven-blade beta-propeller surrounded by a-helices and is in agreement with previously reported theoretical models of SBP1 from *Arabidopsis thaliana* [14] and *Homo sapiens* [48]. In the case of *At*SAH7, its predicted model mainly consists of a seven-stranded β-barrel and closely resembles the protein Pla l 1 from *Plantago lanceolata*, a member of the Ole e 1–like protein family whose structure is experimentally determined [49]. Protein–protein interface residues that were provided as an input to the HADDOCK2.2 Web Server were predicted by the CPORT algorithm. The best solution of the driven docking experiment (Figure 5a) exhibited a HADDOCK score of −102.7 (+/−7.6) and a Z-score of −2.4, with the values of energies (Kcal/mole) having been calculated as follows: [a] van der Waals energy: −68.3 (+/−3.1); [b] electrostatic energy: −348.2 (+/−25.1); [c] desolvation energy: 3.1 (+/−4.9); [d] restraints violation energy: 321.5 (+/−99.64) and [e] total buried surface area (BSA): 2342.8 Å2 (+/−294.8). The high value of the restraints violation energy is the result of the fact that the HADDOCK did not take into account all the residues that were predicted as active by the CPORT algorithm during the docking experiment. The resulting complex was analyzed with PISA software and the residues that are located at the interface between *At*SBP1 and *At*SAH7 are listed in Table 1. The calculations from PISA indicate that the complex formed after the docking experiment is stabilized by a network of 19 hydrogen bonds and three salt bridges between residues that are located at the interface of the proteins (Table 2).

To further shed light on the interaction between *At*SBP1 and *At*SAH7, molecular dynamics simulations were carried out for 200 ns at 300 K utilizing GROMACS. After the completion of the simulation, the derived complex (Figure 5b) was also analyzed with PISA and the residues that were located at the interface between *At*SBP1 and *At*SAH7 are listed in Table 1. Calculations regarding hydrogen bonds and salt bridges were also conducted after that step, and the participating residues in those types of interactions are listed in Table 2. In the last frame of the molecular dynamics simulation, PISA calculated 12 hydrogen bonds and six salt bridges.

As is shown in Table 1 and Table 2, the residues of *At*SBP1 that interact with *At*SAH7 and participate in the formation of hydrogen bonds and salt bridges stabilizing the complex are in good agreement with the results from the yeast two-hybrid (Y2H) assays.

### 2.6. AtSBP1 Interacts in Planta with AtSAH7

**Figure 6:**
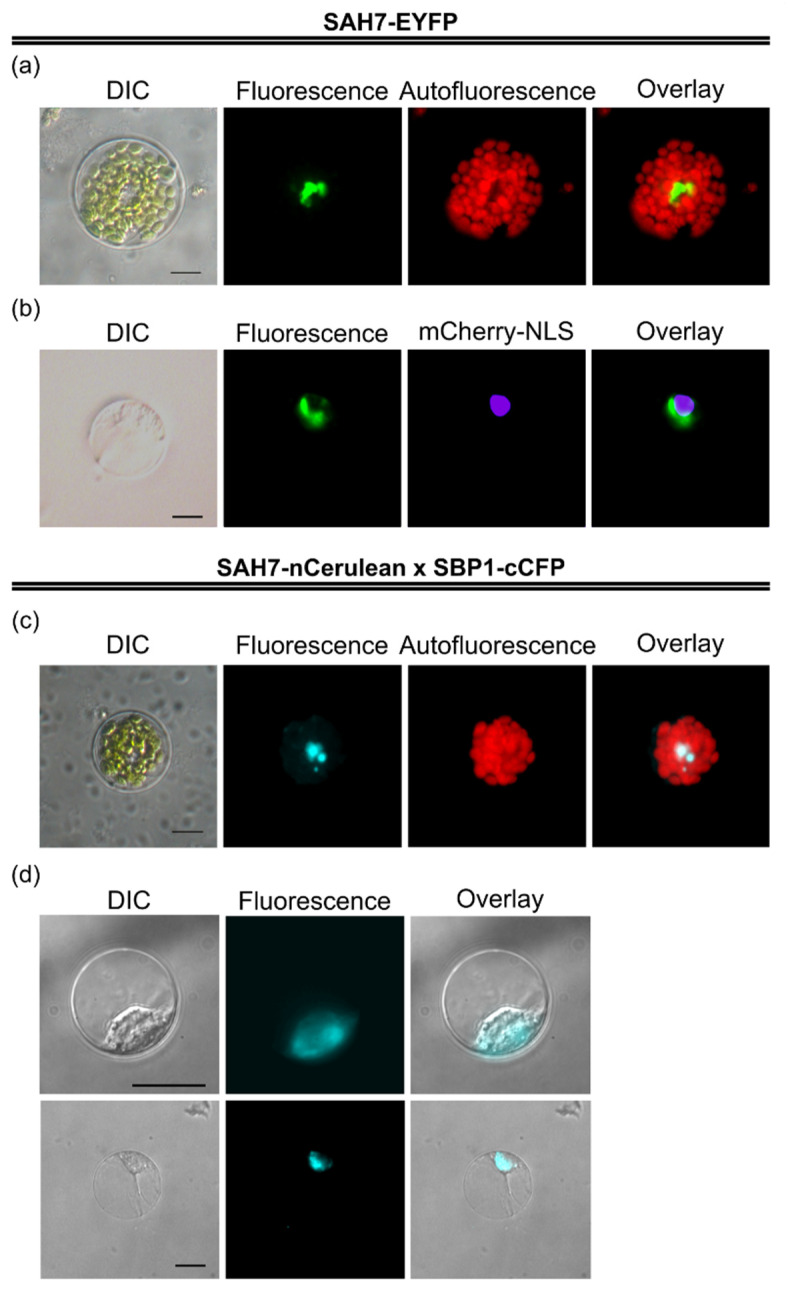
Transient expression of *At*SAH7 and BiFC assays in Arabidopsis mesophyll and root protoplasts. *At*SAH7 was fused to EYFP to study its subcellular localization in mesophyll protoplasts (**a**) and root protoplasts (**b**). Root protoplasts were isolated from stable 35S:mCherry-NLS 10d seedlings. Chimeric constructs of *At*SAH7-nCerulean and *At*SBP1-cCFP generated in order to determine the subcellular localization of the interaction in mesophyll protoplasts (**c**) and root protoplasts (**d**). Fiji (ImageJ) was used in order to pseudocolor mCherry-NLS to magenta (**b**) and BiFC to Cyan (**c**,**d**). *At*SAH7 is localized in a structure around the nucleus, probably ER. *At*SAH7 interacts in planta with all *At*SBP1 in similar structure to the one that *At*SAH7 localizes in. Bars 10 μm.

In order to confirm the in planta interaction of *At*SBP1 with *At*SAH7, we employed bimolecular fluorescence complementation (BiFC) in living protoplast cells [50,51]. For this analysis, *At*SBP1 was fused to cCFP and *At*SAH7 to nCerulean. cCFP and nCerulean are not able to emit fluorescence themselves, but only under close proximity. This approach was carried out in mesophyll protoplasts as well as in root protoplasts, and the fluorescent signal of the interaction was restricted in a horseshoe structure surrounding the nucleus, like *At*SAH7-EYFP (Figure 6), indicating that *At*SAH7 interacts with *At*SBP1 in the ER.

The complex of *At*SAH7 and *At*SBP1 in the ER could imply a possible role in the post-translation modification of other proteins. According to previous studies of auxin accumulation in the ER, we cannot exclude a hypothesis of the *At*SAH7 and *At*SBP1 complex being part of the regulation pathway of auxin.

Based on the research of Ozgur and colleagues, ER stress stimulates ROS production and signaling, conducts changes in redox state and regulates the antioxidant defense [52]. Moreover, previous studies revealed that *At*SBP1 is a redox regulator. Thus, we hypothesize that the interaction of *At*SAH7 with *At*SBP1 may induce the production of antioxidants and promote the appropriate defense responses.

## 3. Materials and Methods

### 3.1. Plant Material and Growth Conditions

All *Arabidopsis thaliana* plants were Columbia (Col-0) ecotype and used for floral dip transformation mediated by *Agrobacterium tumefaciens* strain GV3101 [53]. Six growing pots, each containing five Arabidopsis plants, were used for each construct. Seeds were initially stratified for two days at 4 °C, surface sterilized for two min in 75% (*v*/*v*) ethanol, and 4 min in 25% (*v*/*v*) bleach, and washed with sterile distilled water. Afterwards, they were transferred to soil (Postground P, Klasmann-Deilmann, Geeste, Germany) or plated on Petri dishes containing solid half-strength MS medium (Duchefa Biochemie, Haarlem, The Netherlands) [54] supplemented with 0.05% MES (2-(N-morpholino) ethanesulfonic acid) (Sigma-Aldrich, St. Louis, MO, USA) pH 5.7, Gamborg’s B5 vitamins, a micronutrient mixture (Duchefa Biochemie, Haarlem, The Netherlands), 2% (*w*/*v*) sucrose and 1.2% (*w*/*v*) agar (Difco Laboratories, Detroit, MI, USA). The seeds were germinated and grown in a plant growth chamber under long-day conditions at 22 °C (16 h photoperiod).

### 3.2. Construction of Vectors

For promoter analysis, genomic DNA was used for the amplification of the promoter sequence of *At*SAH7 (−3435 bp). The set of oligonucleotides used for the amplification was designed based on the nucleotide sequences available in the TAIR database for the accession number AT4G08685, and properly modified to include unique restriction sites. The primers used are presented in Table S2. For the amplification, LongAmp Taq DNA Polymerase (New England Biolabs, Beverly, MA, USA) was used. This PCR product was first cloned in pJET1.2 vector (Thermo Sientific™, Waltham, MA, USA) and then in AgeI/NcoI sites of pCambia-1301 binary vector in order to control the expression of the β-glucuronidase (GUS) reporter gene. pJET1.2/pSAH7 was used for the amplification and creation of three 5′-deletions (pSAH7 Δ1 (−1420 bp), pSAH7 Δ2 (−993 pb)) and cloned in BamHI/NcoI restriction sites in pCambia-1301.

cDNA from 10 day Arabidopsis seedlings served as a template to amplify *AtSAH7* CDS (AT4G08685) using gene-specific primers (Table S2). *AtSAH7* was first cloned in pJET1.2 vector (Thermo Scientific™, Waltham, MA, USA) and then in pGBKT7 and pGADT7 (Clontech, Mountain View, CA, USA) with NdeI/BamHI restriction sites. Similarly, *AtSBP1* (AT4G14030), *AtSBP2* (AT4G14040), *AtSBP3* (AT3G23800), and the deletions of *AtSBP1*(DEL1-8) from the full length *AtSBP1* cDNA were cloned in the NdeI/BamHI sites of the pGADT7 vector, except *AtSBP1*DEL8 that was also cloned in pGBKT7 in the same restriction sites [16].

Stable Arabidopsis plants with nucleus localization were created by Dervisi et al. [18].

For the localization study, *AtSAH7* was amplified from pJET1.2/SAH7 using specific primers (Table S2). This product was cloned in the EcoRI/BamHI restriction sites of pSAT6-EYFP-N (pE3225, CD3-1104) [55]. The 35S:SAH7-EYFP cassette was cloned to the binary vector pPZP-RCS2-ntpII (pE3184, CD3-1061) [55] in the PI-PspI restriction site.

For the bimolecular fluorescence complementation assay, appropriate vectors were used [51]. pJET1.2/SAH7 was also used for its amplification with specific primers (Supplementary Table S2) and then cloned in pSAT4-nCerulean-C (pE3416, CD3-1090) with EcoRI/BamHI restriction sites. *AtSBP1* was cloned in pSAT1-cCFP-N1 (pE3449, CD3-1069), as described by Valassakis et al. [16].

### 3.3. Chemical Treatment of Plants

Arabidopsis seeds plated on half-strength MS medium were grown vertically for 4 days. At this point, young seedlings were transplanted onto plates containing half-strength MS medium plus 150 μM selenite (Na_2_SeO_3_; Sigma-Aldrich, St. Louis, MO, USA), 150 μM sodium selenate (Na_2_SeO_4_; Alfa Aesar, Karlsruhe, Germany) and 150 μM cadmium chloride (CdCl_2_; Fluka Honeywell International Inc., Charlotte, NC, USA) and grown under the conditions mentioned above for 4 days. The chemicals used for the treatments were maintained in 50 mM stock solutions in distilled water. Seedlings transplanted onto plates containing only half-strength MS medium were used as controls. Roots from 8 day old control and treated seedlings were collected, weighted to 100 mg, frozen in liquid nitrogen and stored in −80 °C for use in real-time quantitative RT-PCR analysis. In total, three biological replications were performed.

### 3.4. Stainings

GUS histochemical analysis was performed in 7-day seedlings. For GUS staining, the samples were prefixed in 90% acetone for 30 min at 4 °C and washed twice with 100 mM sodium phosphate buffer pH 7.0 containing 0.1 M K_3_Fe(CN)_6_, 0.1 M K_4_Fe(CN)_6_ and 10% (*v*/*v*) Triton X-100. The samples were then incubated at 37 °C overnight in a reaction buffer containing 0.9 mg ml−1 5-bromo-4-chloro-3-indolyl β-D-glucuronide sodium salt as substrate (Melford Laboratories Ltd., Ipswich, England) in the same buffer [56]. Samples were cleared in graded ethanol series (20%, 35%, 50%, 70% 90%, 100% *v*/*v*) for 30 min each and immersed in 90% (*v*/*v*) ethanol for 30 min. Finally, they were kept overnight at room temperature in an aqueous chloral hydrate clearing solution containing glycerol and stored in the same solution. Fifteen to twenty independent T2 lines were stained for each construct. More than 10 seedlings were examined from each line.

To determine the cellular expression of the allergen, we followed a Propidium Iodine (PI, SERVA Feinbiochemica Gmbzh & Co., Heidelberg, Germany) and Hoechst 33258 (Sigma-Aldrich, St. Louis, MO, USA) staining in 7-day-old plants, as described by Benfey Lab and Moller and McPherson [57], respectively. In more detail, seedlings were dipped in PI for 1 min, washed in distilled water and subsequently dipped in Hoechest solution for 5 min, followed by washes and observation in confocal microscope. Fifteen to twenty independent T2 lines were stained for each construct. More than 10 seedlings were examined from each line.

### 3.5. Yeast Two-Hybrid Assays

Yeast co-transformation was based on the lithium acetate method (Matchmaker^TM^ Gold Yeast Two-Hybrid User Manual, Clontech, TaKaRa, Dalian, China). The *Saccharomyces cerevisiae* strain was Gold Y2H (MATa, trp1-901, leu2-3, 112, ura3-52, his3-200, gal4Δ, gal80Δ, LYS::GAL1_UAS_-GAL1_TATA_-Ade2, URA3::MEL1_UAS_-Mel1_TATA_AUR1-C MEL1) and grown in YPDA medium. For transformant selection a synthetic dropout medium/plate (SD medium) lacking leucine and tryptophan (SD-Leu-trp) was used. Interactions were checked in SD medium without leucine, tryptophan, histidine and adenine (SD-Leu-Trp-His-Ade). More than 5 independent colonies were tested.

### 3.6. Structural Prediction of Protein–Protein Interactions by Molecular Docking and Molecular Dynamics Simulations

Prediction of protein–protein interactions between SBP1 and SAH7 from Arabidopsis thaliana were carried out via molecular docking and molecular dynamics simulations. The molecular docking experiment was performed by the application of the “Prediction Interface” of the HADDOCK2.2 Web Server (accessed on 23 October 2022) [58,59]. The 3D structures of *At*SBP1 and *At*SAH7 used in the docking experiment were retrieved from AlphaFold Protein Structure Database (Alphafold DB, accessed on 10 October 2022) that is the result of the collaboration between DeepMind and EMBL’s European Bioinformatics Institute (EBI) [60,61]. Alphafold is an Artificial Intelligence system that predicts the 3D structure of a protein from its amino acid sequence, achieving accuracy comparable to experimental data. The latest release of Alphafold DB provides structural models for almost all the entries of Uniprot [62], and since there are no available experimental structural data for *At*SBP1 and *At*SAH7 we retrieved their structural models from the Alphafold database (Uniprot AC numbers of *At*SBP1 and *At*SAH7 are O23264 and Q9SZY5, respectively). In the case of *At*SBP1, residues 1–18 were not taken into account due to a low per-residue confidence score (pLDDT) as it is calculated by Alphafold, implying low quality data for that part of the model. As far as *At*SAH7 is concerned, coordinates of residues 1–19 were also not used in the docking experiment because they correspond to a signal peptide as predicted by SignalP (accessed on 12 October 2022) [63].

The protein–protein interface residues that were provided as input to the HADDOCK2.2 Web Server were predicted by the CPORT algorithm (accessed on 23 October 2022) [64]. The generated poses were ranked by the HADDOCK score, that is, the weighted sum of inter-molecular electrostatic (Eelec), van der Waals (EvdW), desolvation (ΔGsolv) and ambiguous interaction restraint (AIR) energies.

Molecular dynamics simulations were performed on the complex that exhibited the highest HADDOCK score with the use of GROMACS software suite v. 2018.1 [65] and the employment of the AMBER99SB-ILDN force-field [66]. The complex was placed in a 1.2 nm cubic box containing 3-point model (TIP3P) water [67], while neutral pH conditions were achieved by the addition of NaCl molecules. The system was subjected to energy minimization (maximum of 2000 steps) using the steepest descent algorithm. Two phases of equilibration took place with position restraints applied on protein coordinates. First, a simulation was performed for 100 ps under a constant volume (NVT) ensemble to equilibrate temperature at 300 K, using the Berendsen thermostat [68]. A second equilibration for 100 ps was performed in the isothermal–isobaric (NPT) ensemble to control pressure isotopically at 1.013 bar (1 atm), using the Berendsen weak coupling algorithm [69] and the Berendsen thermostat at 300 K. Finally, the equilibrated complex was subjected to a molecular dynamics simulation, with position restraints removed, for 200 ns at 300 K. Periodic boundary conditions were applied in all directions. Bond constraints were modeled with the application of the LINCS algorithm [70] and the use of a 2 fs time-step. Short-range non-bonded interactions were modeled using a twin-range cutoff at 1.0 nm, while the Particle Mesh Ewald (PME) method was used for the modeling of long-range electrostatic interactions, with Fourier grid spacing at 0.16 nm [71].

The properties of the interfaces of the interacting proteins were calculated utilizing PISA software (accessed on 24 October 2022 and 10 November 2022) [72]. The resulting models were visualized with the PyMol Molecular Visualization System [73].

### 3.7. Protoplast Analysis

For the isolation and transformation of mesophyll protoplasts we followed the Tape-Arabidopsis Sandwich method, as described by Wu and colleagues [74], while for the isolation of root protoplasts we used the protocol of Bargmann and Birnbaum [75]. Three independent repetitions were performed.

### 3.8. Microscopy

Samples were examined with a Zeiss Axioplan fluorescence microscope (Zeiss, Oberkochen, Germany) equipped with a differential interference contrast (DIC) optical system and an Axiocam MRc5 digital camera (Zeiss). For fluorescence images the same exposure time was used. Moreover, a Zeiss Stemi 2000-C stereomicroscope equipped with a Jenoptik ProgRes3 (Jenoptik, Jena, Germany) digital camera was also used.

### 3.9. Confocal Microscopy

Specimens were examined on a multiphoton inverted confocal microscope (Leica TCS SP8 X, Wetzlar, Germany) equipped with a UV (laser) and a White Light Laser (WLL). For the detection of EYFP, Hoechst/Lignin and PI excitation peak centered at 490 nm, 406 nm and 538 nm, respectively, the emitted light was captured at 495−570 nm for EYFP, 422–485 nm for Hoechst/Lignin and at 600–650 nm for PI by a HyD and a PMT detector. Acquisition was performed with the Application Suite X (LAS X) (Leica Microsystems CMS GmbH, Wetzlar, Germany) using the same parameters for all specimens.

### 3.10. RNA Extraction, cDNA Synthesis and Gene Expression Analysis

Total RNA was isolated RNA following the procedure described by Oñate-Sanchez and Vicente-Carbajosa [76]. In more detail, we collected 100 mg tissue of 3-day-old seedlings, 10-day-old seedlings, 10d root, 10d cotyledons and 10d shoots as well as of treated roots. One whole rosette leaf and 8 flowers were collected. RNA samples were treated with DNAse I (New England Biolabs, Beverly, MA, USA) according to the manufacturer’s instructions. First-strand cDNA synthesis was performed using 1 μg of total RNA, oligodT primers and SMART MMLV RT (Takara-Clontech, Kyoto, Japan). For quantitative (q) RT-PCR, KAPA SYBR^®^ FAST qPCR Master Mix (2×) Kit (Kapa Biosystems, Woburn, MA, USA) was used according to the manufacturer’s instructions. The reactions (total volume 10 μL) were performed in a thermal cycler (Applied Biosystems, Foster City, CA, USA). Ubiquitin 10 (UBQ10) was used as housekeeping control for normalization. Gene expression experiments were performed in three biological replications and technical triplicate. The relative expression levels of target genes and SD values were calculated using the 2^−ΔΔCT^ Livak method [77], and statistically significant differences in expression between samples were detected using a t-test. Those with *p* < 0.05 were considered statistically significant. SigmaPlot statistical software (Version 10.0, Systat Software Inc., Richmond, CA, USA) was used to analyze statistical significance. Variance analysis was performed using one-way ANOVA with Tukey’s HSD test.

### 3.11. Imaging

Images were edited via Fiji (NIH, Bethesda, MD, USA) [78] and the final figures were generated by Inkscape (Version 1.2.2, Software Freedom Conservancy Inc., Brooklyn, NY, USA) [79].

## 4. Conclusions

The allergen *At*SAH7 is expressed in the vascular tissues of leaves and roots where, at the sub-cellular level, it is localized in the root xylem and the ER of the root phloem and root hairs, indicating its importance in root development. The promoter fragment pSAH7 Δ1 is capable of acting as its functional promoter. Moreover, *At*SAH7 participates in regulating responses to selenite and probably plays a role in oxidative stress. Our results showed that *At*SAH7 interacts with *At*SBP1, most likely in the ER, and that the first 105 aa of *At*SBP1 are important for this interaction. The lack of literature and experimental information on *At*SAH7 allows us to form many hypotheses. The probable correlation of *At*SAH7 with ABA and ROS and the expression of *At*SBP1 in guard cells implies a role of the interaction *At*SBP1/*At*SAH7 in stomatal closure under stress. A possible role of the *At*SBP1 and *At*SAH7 complex can be the promotion of antioxidant production and the activation of defense responses triggered by ER stress.

## Figures and Tables

**Table 1:** List of the residues that are located at the interface between *At*SBP1 and *At*SAH7 after the molecular docking experiment and after the molecular dynamics simulation. (A) Interfacing residues of *At*SBP1 after the docking experiment, (B) interfacing residues of *At*SBP1 after the MD simulation, (C) interfacing residues of *At*SAH7 after the docking experiment, (D) interfacing residues of *At*SAH7 after the MD simulation.

A	B	C	D
19–21, 23–25, 30–31, 33–34, 36–40, 68–69, 99–106, 160–161, 185–186, 191, 194, 196, 211–212, 214, 250,252, 254, 277, 412–413, 415–416	19–23, 30–31, 99–106, 137, 159–161, 163, 183, 185–187, 191, 193–196, 250,252	34, 40–45, 47, 49, 62–65, 90–91, 93, 95, 111–119, 121, 123–126, 130–135, 151, 154, 156–159	42–45, 47, 49, 61–63, 65, 90–93, 112–113, 115–121, 123–124, 131–135,159

**Table 2:** List of *At*SBP1 and *At*SAH7 residues that participate in the formation of hydrogen bonds and salt bridges after the molecular docking experiment and after the molecular dynamics simulation. (A) *At*SBP1 (resulting complex of the docking experiment), (B) *At*SBP1 (last frame of the MD simulation), (C) *At*SAH7 (resulting complex of the docking experiment), (D) *At*SAH7 (last frame of the MD simulation).

	A	B	C	D
Residues that participate in hydrogen bonds	Gly20, Lys23, Tyr24, Gly25, Thr31, Ser40, Gly102, Asp103, Pro251, Lys415	Lys19, Cys22, Gly102, Asp103, Ala104, Ser105, Glu163, Trp194, Gly252	Tyr34, Gly41, Glu43, Thr44, Ser47, Arg62, Thr119, His113, Asp114, Thr119, Asn124, Phe132, Asn134, Asn135, Tyr154, Glu156, Glu158	Glu43, Pro45, Arg62, Arg63, Arg117, Thr119, Arg131, Phe132, Asn134
Residues that participate in salt bridges	Lys23, Arg212, Lys415	Lys19, Glu163	Glu43, Asp114, Glu158	Glu43, Arg62, Asp159

## Data Availability

All data supporting the findings of this study are available within the paper and within its Supplementary Materials published online.

## Supplementary Materials

The following supporting information can be downloaded at: https://www.mdpi.com/article/10.3390/ijms24043580/s1.
