# Supplementary material for: The SAH7 Homologue of the Allergen Ole e 1 Interacts with the Putative Stress Sensor SBP1 (Selenium-Binding Protein 1) in Arabidopsis thaliana
Source: Int J Mol Sci. 2023 Feb 10;24(4):3580. doi: 10.3390/ijms24043580 (PMC9962204; doi:10.3390/ijms24043580)
Supplement: Supplementary file 1 [file ijms-24-03580-s001.zip › ijms-2161615-supplementary.pdf]

Table S1. Regulatory promoter elements in pSAH7 Δ2 (-1420 bp to -993 bp) from the PlantCARE database.

Motif	Position (bp)	Strand	Sequence	Function
GT1-motif	-1221, -1219,	-, +	GGTTAA	Light responsive element
HD- Zip 1	-1237	+	GAAT(A/T)A TTG	Element involved in differentiation of the palisade mesophyll cells
Myc	-1035	+	TCTCTTA	Abscisic acid responses, drought
TCT-motif	-1396	+	TCTTAC	Light response

Table S2. Sequence of primers used in the present study

Name	Sequence (5′ ->3′)
SAH7-F-RI	GAATTCATGTCTAAAGCAGTTCTATTGGTCGC
SAH7-R-BS	TACTAGTGGGGGGATCCCGTCCTCGGTTTCTTGGTATAGC
SAH7-F-NN	CGCGGCCGCCATATGTCTAAAGCAGTTCTATTGGTCGCTC
SAH7-R	CGTCCTCGGTTTCTTGGTATAG
SAH7-RLT-F	TCTCCGGTGCAGTGGTTAGA
SAH7-RLT-R	ATGACCAGGGGACACATTGG
pSAH7-F-AgeI	TACCGGTGTGTTTGGGCTATATTTAATTGGCTGCTTG
pSAH7-F2-BamHI	TGGATCCGGTAGCTAGGATTTTTGAAACAAATCGGAAG
pSAH7-F3-BamHI	AGGATCCGCACAATAAAAGGGAATTAGACTTTTTC
pSAH7-F4-BamHI	AGGATCCGTTTAACCCATTTTTGATGTTCATAGTGAGC
pSAH7-R2-NcoI	GACCATGGTGCAAAAGATTTTAACTGGAAAAG
